# Open Science practices in a Portuguese nursing higher education institution: An exploratory, descriptive study

**DOI:** 10.1371/journal.pone.0322597

**Published:** 2025-10-16

**Authors:** Ana Sabrina Sousa, Mafalda Lopes, Diana Rodrigues, Palmira Oliveira, Regina Pires, Sara Pinto, Pedro Melo, João Frias Rosa, Amparo Alves, Rosa Silva

**Affiliations:** 1 RISE-Health, Nursing School of Porto, Porto, Portugal; 2 Nursing School of Porto (ESEP), Porto, Portugal; University of Coimbra: Universidade de Coimbra, PORTUGAL

## Abstract

Open Science has emerged as an epistemological paradigm that promotes open and dynamic access to knowledge, fostering the sharing of scientific findings, collaboration between academia and society, and enhancing innovation and transparency. However, comprehensive studies on the adoption of Open Science practices remain limited, particularly in the nursing academic context. This study aimed to identify the current Open Science practices within a Portuguese higher nursing education institution community. An exploratory, descriptive study was conducted in two phases. In the first phase, a questionnaire was adapted and piloted through a focus group. In the second phase, the final version of the questionnaire, with 34 questions, was distributed digitally to 747 teachers, researchers and students. The study included 35 respondents, predominantly faculty members. While 77.8% reported engaging with Open Science publications, fewer respondents demonstrated familiarity with practices such as study preregistration and open-source coding. The principles of Open Science were widely accepted, particularly those emphasizing ethics, the democratization of knowledge, and scientific collaboration. Participants frequently used tools like EBSCOhost, Medline, and Scopus, whereas platforms such as Zenodo and OpenUP Hub remained underutilized. Participants highlighted training priorities, especially in open access publishing, data management, and the implementation of Open Science recommendations. The low response rate may indicate limited awareness of Open Science among professionals and an institutional culture in which such practices have not been fully embedded within academic routines. Although all participants agreed with the principles of Open Science, most reported not knowing its policies and not all were familiar with the various formats. These findings underscore the importance of investing in skills training and raising awareness of Open Science practices, particularly within the nursing school context. Furthermore, they underscore the need for in-depth investigation into the motivations, barriers, and enabling conditions that influence adherence to Open Science in higher education.

## Introduction

As an emerging epistemological paradigm, Open Science (OS) is reshaping the way knowledge is shared, fostering openness, reproducibility, and closer interaction between researchers and society [1,2]. Grounded in the principles of findability, accessibility, interoperability, and reusability (FAIR), OS promotes innovation while maximizing the scientific, social, and economic impact of research outputs [3–5]. Over the past decade, the OS ecosystem has expanded considerably, reflecting increasing adoption across disciplines and institutions and reinforcing its transformative potential in the production, validation, and dissemination of scientific knowledge [6,7]. International organizations, such as the OECD, emphasize the benefits of OS, such as reduced research costs, wider reuse of data, improved transparency and accountability, accelerated knowledge transfer, and greater citizen participation in scientific endeavors [8].

The core principles of OS encompass methodological transparency, open availability and reuse of research outputs, and the use of digital tools to facilitate collaboration [1,9]. These principles are operationalized through practices such as open access publishing, data sharing, open-source software development, reproducible methodologies, and citizen science initiatives [3,10,11]. While such practices are increasingly embedded in science policy frameworks, their implementation within day-to-day research activities remains uneven, and barriers persist at both institutional and professional levels, particularly regarding awareness, training, and infrastructure [7,12].

Given its growing importance, higher education institutions (HEI) are urged to adopt proactive strategies to advance OS and maximize its benefits [13]. However, the existing body of scientific evidence on this topic remains limited. Comprehensive studies that systematically map current OS practices within the academic community encompassing diagnostic analyses of these practices, levels of knowledge, and the perceived stakeholders’ needs are still scarce, especially in the nursing field. In this context, nursing schools, as integral components of the Portuguese higher education system, play a pivotal role in preparing healthcare professionals and generating applied scientific knowledge. Despite this relevance, no studies were identified that specifically address OS practices within Portuguese higher education nursing schools.

A preliminary qualitative study conducted with Portuguese health researchers further highlights this gap, revealing that the emerging terminology and discourse surrounding OS often generate conceptual ambiguity [14]. Researchers display heterogeneous behaviors toward scientific information, influenced by factors such as their (in)experience, institutional guidelines, individual judgments of what is most reasonable, and individual interpretations of national and European policies and recommendations. These findings underscore the need for systematic studies capable of diagnosing existing practices, assessing levels of awareness, and identifying the support structures required to strengthen OS adoption in specific institutional contexts.

In light of these considerations, the present study aimed to identify the current OS practices within a Portuguese higher nursing education institution community and, consequently, to assess its training and infrastructure requirements. By addressing this gap, the study provides institution-specific evidence that may inform the design of targeted strategies to foster OS within nursing education and research, thereby strengthening the broader promotion and implementation of open and transparent science practices across higher education.

## Methodology

To ensure transparency and reproducibility, this study adhered to the guidelines established by the Strengthening the Reporting of Observational Studies in Epidemiology (STROBE) [15] throughout all stages, from study design to data analysis.

### Study design

An exploratory, descriptive study was conducted at a public Portuguese nursing HEI. The study design was structured in two main phases, which are presented below.

### Questionnaire adaptation

The questionnaire employed in this study was developed based on two main sources: the Marques et al. [16] questionnaire and the Science Europe Principles on Open Access to Research Publications [17]. These references provided the conceptual and methodological basis for item formulation and refinement, thereby ensuring alignment with previous research and international guidelines. The adaptation process was led by a team member with recognized expertise in the field under study (ML). Following this process, the questionnaire was reviewed by two external experts (FF, JA), who suggested minor adjustments.

Subsequently, the questionnaire was piloted through a focus group with 10 participants in January 2024, including postgraduate students and faculty members. These participants were selected through convenience sampling from the research team’s network of contacts. This phase aimed at evaluating the content validity, appropriateness, and applicability of the questionnaire.

Three members (ML, DR, RS) of the research team participated in the focus group sessions, documenting all relevant information. The focus group reviewed the entire questionnaire, and changes were implemented based on the majority consensus. In instances where a definitive conclusion could not be reached, the information discussed during the sessions was carefully analyzed by the research team, and additional adjustments were made based on a consensus-driven approach.

### Questionnaire application

The final version of the questionnaire [18] was made available on a digital platform and distributed to the target population, which included faculty members and postgraduate students at the HEI, excluding undergraduate. The questionnaire comprised 34 questions with multiple-choice items and open-ended fields for free-text answers.

The data were gathered from 1^st^ February to 31^st^ May 2024. The sample represented an approximate population of 747 individuals (members at the institution allocate 30% of their teaching workload to research activities, except for adjunct lecturers). The student body consisted of healthcare professionals, specifically nurses enrolled in postgraduate or master’s programs. The questionnaire was disseminated through the institution’s internal communication channels, including email and newsletters.

### Data collection instrument

The variables examined in this study were organized into four main groups. The first group, **Research Participation**, included aspects such as participation in research groups or centers and the participants’ roles in research projects. The second group, **OS Practices**, focused on indicators like knowledge of OS, participation in OS projects, familiarity with open access policies, and practical experience with OS components, such as publications, educational materials, and data sharing. The third group, **Tools and Platforms**, covered the use of bibliographic databases, institutional repositories, and specialized OS tools. Finally, the fourth group, **Perceptions and Support Needs**, addressed indicators such as the perceived advantages and disadvantages of OS, the main needs for support, and the sources of OS-related support utilized by participants. This information is presented in Table 1.

**Table 1:** Indicators Assessed in the Open Science Practices Questionnaire.

Variables	Indicators
Participant characterization	Participation in Research Groups or Centers Role in Research Projects
OS Practices	Knowledge of OS Participation in OS Projects Familiarity with Open Access Policies Practical Experience with OS Components
Tools and Platforms	Use of Bibliographic Databases Use of Institutional Repositories Use of Specialized OS Tools
Perceptions and Support Needs	Perceived Advantages and Disadvantages Main Needs for Support Sources of OS Support

### Ethical procedures

The study was approved by the Nursing School of Porto ethics committee, ensuring full compliance with the General Data Protection Regulation (GDPR) to ensure anonymity (approval number CE_31/2023) in December 2023.

Participants were informed of the study’s objectives and provided written informed consent before completing the questionnaire. Identification codes were assigned to focus group participants to ensure anonymity and confidentiality (P1, P2,...). Data were securely stored on institutional servers.

### Data analysis

Information from the focus groups and open-ended questionnaire responses were submitted for qualitative analysis. The focus group data were transcribed and subjected to content analysis to identify emerging thematic categories, which were then discussed within the research team. Similarly, the open-ended responses were analyzed to identify relevant patterns and categories, which complemented the quantitative analysis.

Subsequently, a descriptive analysis of the variables was performed using IBM SPSS Statistics V.24 software. Incomplete responses were excluded to ensure the integrity and representativeness of the results. Variable codebook, metadata, and analytic scripts have been deposited in OSF and are openly accessible at: [https://osf.io/8hf75/}.

## Results

### Questionnaire adaptation

**Fig 1:**
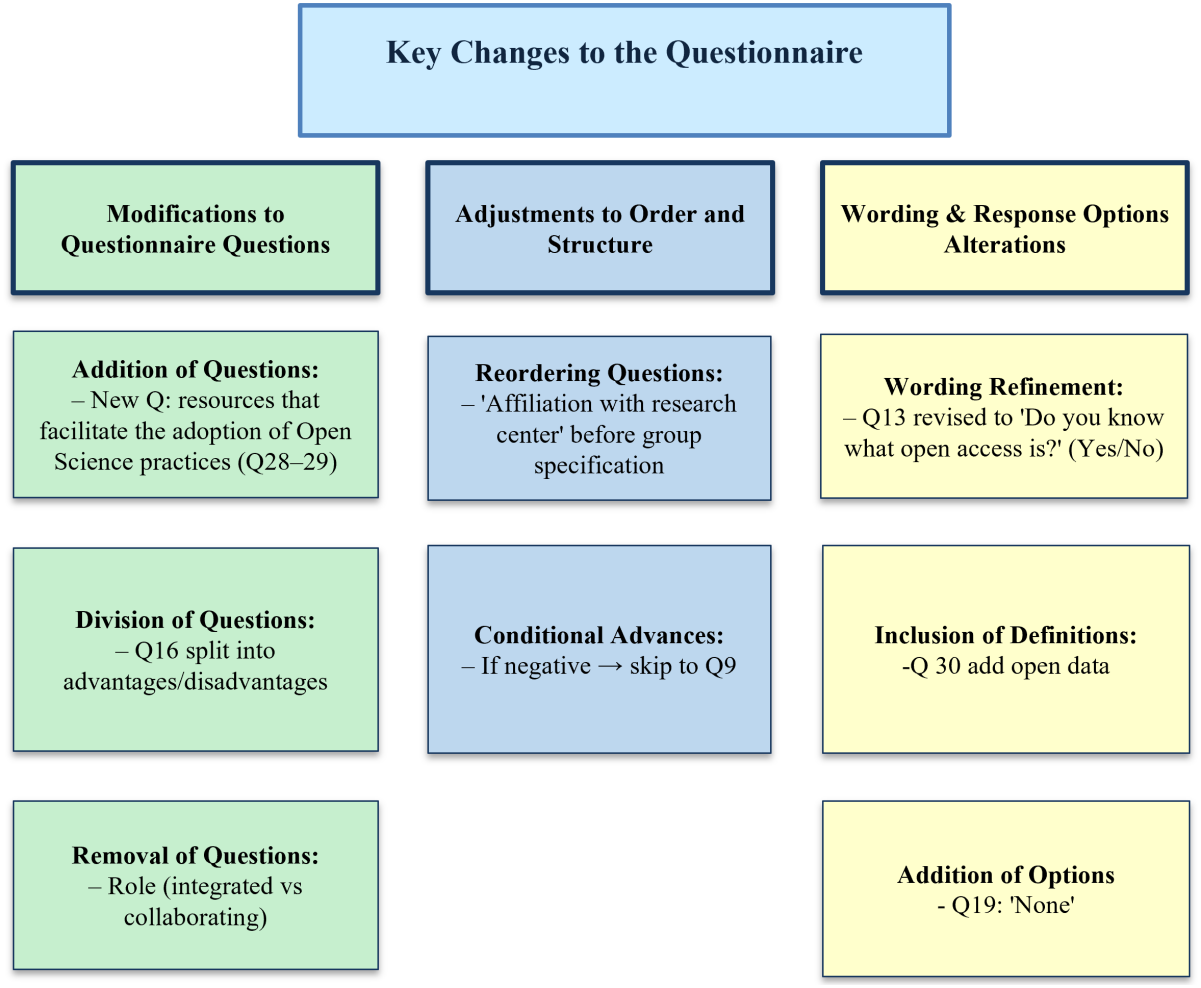
Key changes to the Questionnaire.

The focus group provided valuable insights to refine the questionnaire on OS practices. The modifications were categorized into three main areas: changes to questions, adjustments to the order and structure, and refinements in wording and response options, as detailed in Fig 1. Several new questions were added to the questionnaire to capture additional aspects and broaden the range of possible answers, such as resources that facilitate the adoption of OS practices, while new response options (e.g., “None” and “I have never searched for information”) were added to broaden the range of possible answers. To enhance clarity and data analysis, extensive or complex questions were divided. For example, the original item addressing both the advantages and disadvantages of OS was split into two separate questions. Conversely, items deemed less relevant to the study’s objectives, such as the one regarding whether participants were integrated or collaborating researchers, were removed. The sequence of items was also revised to ensure logical coherence; for example, the question on affiliation with a research center now precedes the question about specifying the research group or center. In addition, wording was refined to improve objectivity, exemplified by the reformulation of a question on familiarity with open access into the direct format: “Do you know what open access is? (Yes/No).” Finally, selected response options were supplemented with definitions and examples to support participant comprehension. Collectively, these adjustments enhanced the clarity, efficiency of the questionnaire and alignment with the study’s objectives, thereby facilitating the collection of more accurate and relevant data.

### Participants characterization

**Fig 2:**
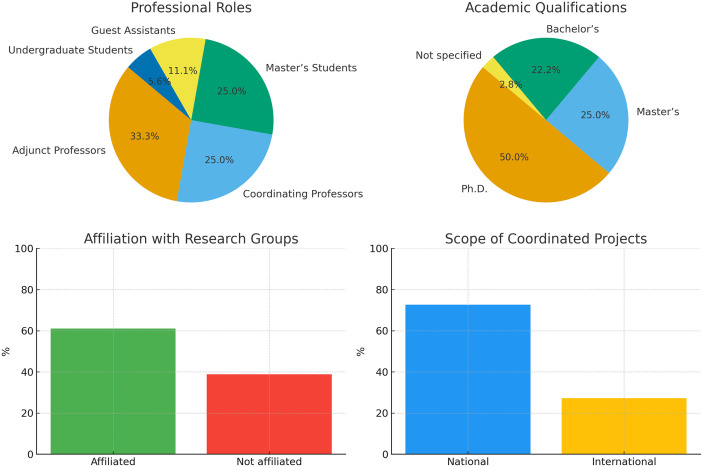
Participants characterization.

The questionnaire was completed by 35 participants from the institution, out of a total population of 747 individuals (faculty and students). The analysis revealed that most respondents held faculty positions, namely Adjunct Professors (33.3%) and Coordinating Professors (25.0%), whereas students represented a smaller proportion of the sample. Half of the participants held a Ph.D., confirming a high level of academic qualification. Furthermore, 61.1% reported affiliation with research groups or centers, mainly as integrated researchers. Regarding project coordination, 30.6% of participants indicated leadership roles, with many of these projects being national in scope (72.7%), although international initiatives were also represented. These results are summarized in Fig 2 and suggest a predominance of projects focused on local contexts but with growing initiatives for large-scale collaboration.

### Open Science practices

**Fig 3:**
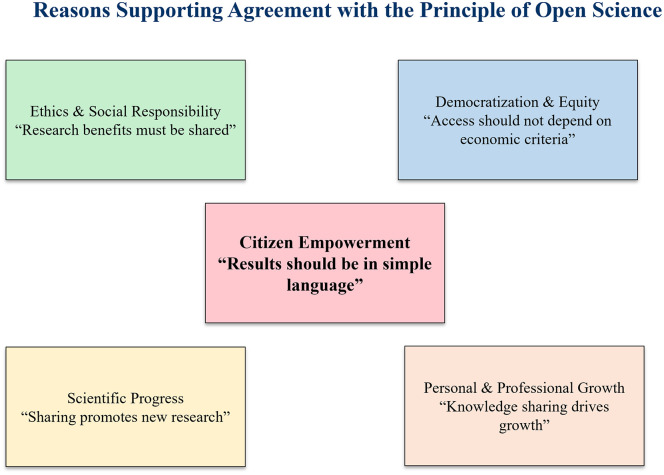
Reasons for supporting agreement with the principle of Open Science.

A key question in the questionnaire examined participants’ agreement with the principle of OS, as expressed in the statement: “Knowledge is for everyone and belongs to everyone”, the results showed unanimous agreement, with 100% of respondents supporting this view. This consensus reflects a strong acceptance of OS core values within the academic community. When asked to articulate their reasons for supporting the principle of OS, participants consistently underscored its ethical and social relevance, framing knowledge sharing as a moral obligation toward human and societal development. They also highlighted its role in democratizing access to knowledge, arguing that economic or social barriers should not determine who benefits from scientific advances. In addition to these ethical considerations, respondents highlighted the contribution of openness to scientific progress, particularly its potential to stimulate collaboration, generate new insights, and expose research gaps. On a more individual level, open practices were associated with personal and professional growth, reinforcing the view that collective and individual development are achievable only through the sharing of knowledge. Notably, a substantial proportion of participants (44%) stressed the importance of communicating research in clear and accessible language, thereby underscoring the central role of OS in empowering citizens and promoting scientific literacy (Fig 3).

**Fig 4:**
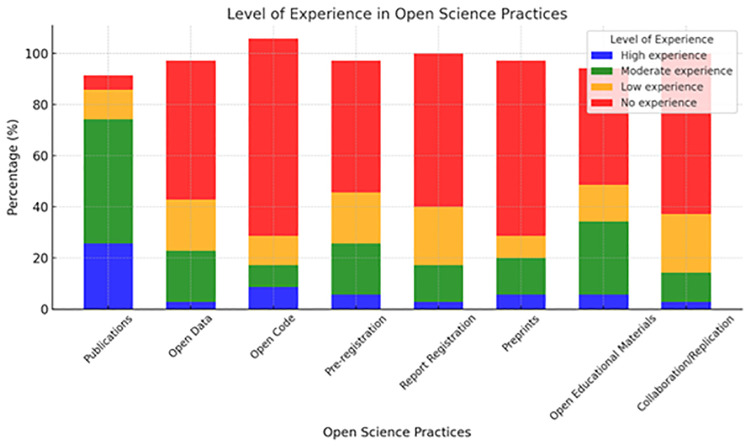
Open Science practices.

Most participants (94.2%) reported having prior experience with at least one of the OS practices mentioned. The most frequently cited practices included open access to scientific publications (94.2%) (e.g., journals and repositories), open educational materials, and collaboration/replication across various locations. Conversely, practices such as pre-registration of studies, report registration, and open code were among the least experienced, suggesting potential unfamiliarity or lower adoption of these initiatives within the HEI context (Fig 4).

When asked about their knowledge of open access policies, 75.0% of respondents indicated familiarity with such policies, while 25.0% stated they were unaware of them. Regarding understanding the open access concept, 63.9% of participants reported they had a good understanding, while 36.1% indicated having only a basic notion.

Most participants (77.1%) reported using or producing open access scientific publications, while 11.4% indicated not engaging with this practice, and an equal proportion (11.4%) expressed uncertainty.

### Tools and platforms

Regarding the use of databases or bibliographic research platforms, the most frequently cited platforms included the Virtual Library of the HEI (n = 31), EbscoHost (n = 35), Medline (n = 32), Scopus (n = 30), and Web of Science (n = 27), demonstrating their central relevance to the participants. Approximately 30% of participants reported using the Institutional Repository, followed by the Open Science Framework (OSF, 15%). Tools such as OpenUP Hub (4.3%), Zenodo (2.1%), and others, such as ARGOS/OpenDMP (2.1%) were mentioned less frequently.

### Perceptions and support needs

When asked about the perceived advantages and disadvantages of adopting OS practices, participants identified several key benefits. The main advantages included increased visibility of publications and data, easier access to knowledge, and the promotion of scientific collaboration. However, participants noted significant disadvantages, including time constraints, the undervaluation of OS practices within scientific evaluation systems, and the need to adapt to new methodologies and tools. These challenges represent barriers that must be addressed to successfully implement and consolidate OS practices.

**Fig 5:**
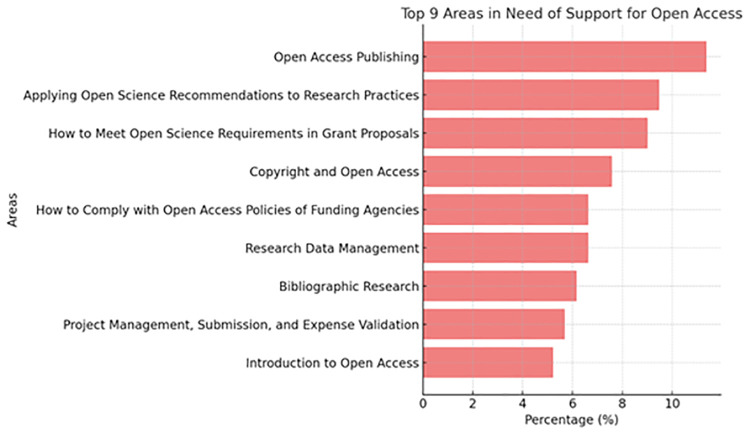
Support Needs for Open Access.

The results indicate that participants identified the greatest need for support and information on Open Access Publishing (11.4%), followed by Applying OS Recommendations in Research Practices (9.5%) and Meeting OS Requirements During Grant Proposal Preparation (9.0%). As summarized in Fig 5, these findings highlight the importance of these areas for the study population.

Among the most frequently mentioned support actions, the publication of educational materials in open access was the most prominent, cited by 77.14% of participants, highlighting the importance of freely sharing educational resources. Additionally, 74.29% of respondents indicated the need for technical guidance on preparing data for deposit in open repositories, reflecting the growing recognition of the importance of data transparency and accessibility.

Furthermore, 60% of participants mentioned preparing digital materials and software code for deposit in open access, demonstrating significant interest in the dissemination of technological resources. Similarly, 60% reported the need for support in the preregistration of high-quality protocols, reinforcing the importance of reproducibility and scientific integrity.

## Discussion

The Foundation for Science and Technology, I.P. (FCT), an agency under the Ministry of Education and Science of Portugal responsible for evaluating and funding scientific research activities across the country in all scientific areas, adopted the Policy on Open Access to Scientific Publications in 2014 for publications resulting from R&D Projects Funded by FCT. However, despite these advancements, such practices are not currently implemented in some contexts. The results of this study offer an exploratory perspective on OS practices within a Portuguese nursing HEI, a context that remains underexplored in the literature. Despite extensive dissemination and strategies implemented by the research team to encourage participation, including institutional communication campaigns, engagement of course coordinators, and individual outreach, adherence to the questionnaire remained markedly low. These findings can be attributed to the decision to administer the questionnaire via electronic mail, which, according to Hernandez et al [19] presents a disadvantage. On the other hand, this approach enables reducing bias in responses, as respondents are not influenced by the physical presence of the researcher. In preparing the questionnaire, the research team considered the need for clarity, objectivity, and conciseness in the formulation of questions, ensuring that response tendencies were not introduced and that the length of the questionnaire adhered to recommended guidelines to improve adherence rate and the quality of responses. Beyond methodological aspects, the low participation rate may also reflect structural and motivational barriers rather than methodological shortcomings alone. Studies have shown that limited engagement with OS initiatives may stem from low institutional prioritization of OS practices and insufficient infrastructure or incentives to support them [20,21]. In many HEI, especially in health-related fields, researchers still perceive OS as an abstract or externally imposed concept, rather than an integral part of their academic mission [22]. This cultural inertia may lead to lack of interest or reluctance to engage in initiatives perceived as bureaucratic or misaligned with existing research norms. This was reflected in our findings: although participants expressed broad agreement with the principles of OS, paradoxical results revealed inconsistencies between self-reported practices and awareness of policies. Such contradictions align with Stebbins’s [23] argument regarding the value of exploratory research in under-studied areas, as these early findings can highlight patterns and contradictions that require deeper investigation. Therefore, the lack of responses to the questionnaire may indicate unfamiliarity with OS practices and a perceived lack of relevance or urgency associated with the topic.

Scholars have emphasized the importance of a situated approach to OS adoption, attentive to local contexts and disciplinary differences [21]. In the present study, limited adoption of practices such as preregistration or open code contrasted with the broader acceptance of open access publishing. Similar results were reported by Ollé et al. [24] in Spanish institutions, where open access was widely adopted, while practices requiring greater methodological transparency were less familiar. Similarly, a recent study of UK psychology doctoral students identified gaps in knowledge and training, particularly regarding preregistration and data sharing [25]. These findings reinforce that awareness of OS does not necessarily translate into comprehensive engagement and that institutional culture strongly influences which practices are more readily adopted.

The cultural dimension of OS has been widely recognized. Vicente-Saez and Martinez-Fuentes [26] argue that OS adoption transcends technical considerations, requiring leadership, collective commitment, and integration into institutional values. In health research, such challenges appear more pronounced. Krahe et al. [27] demonstrated that while researchers value the potential benefits of data sharing, concerns persist regarding privacy, data misuse, and the lack of clear incentives. These insights resonate with our findings, in which participants acknowledged the ethical and social value of openness but expressed hesitation toward practices perceived as complex or misaligned with professional norms.

Awareness and training gaps were also apparent. A lack of literacy in OS – particularly in areas such as data management, preregistration, and licensing – has been identified as a critical barrier to its adoption [28]. The present findings corroborate this view: while participants demonstrated partial familiarity with open access, their knowledge of policies or technical procedures was limited. In Portugal, initiatives such as the OS and Citizen Science at the Polytechnic Institute of Santarém illustrate the importance of embedding OS within institutional strategies, linking openness to social responsibility and community engagement [29]. Such initiatives may serve as models for nursing HEI, where OS remains at an incipient stage. Simultaneously, growing evidence indicates that OS practices, such as preprints and open data, correlate with citation advantages, providing a compelling institutional rationale for promoting broader adoption [30].

Overall, this study highlights that OS adoption within nursing higher education is marked by fragmented practices: while open access publishing is relatively well accepted, other practices remain marginal. This pattern aligns with international findings, suggesting that advancing OS in nursing requires not only infrastructure and training but also cultural change and sustained institutional leadership. Concrete initiatives to support this transition may include the development of research projects specifically oriented toward OS practices, the implementation of training programs for students, the reformulation of curricula to integrate OS into academic units, and the promotion of events such as conferences and workshops dedicated to OS.

The main limitation of this study was the low participation rate, which constrains the generalizability of the findings. However, as suggested by Fecher and Friesike [20], low engagement may itself serve as an indicator of structural and cultural barriers to OS, rather than being attributed solely to methodological shortcomings. This interpretation reinforces the relevance of exploratory studies in under-researched contexts and underscores the need for further investigations using larger samples and mixed-methods approaches.

Future research should build on these findings by examining motivational and institutional factors that influence OS adoption, benchmarking practices across different health-related institutions, and evaluating the effectiveness of training initiatives and policy interventions. Such efforts would support a more comprehensive and inclusive integration of OS in nursing education, thereby contributing to the broader goals of transparency, innovation, and societal impact in science.

By situating these findings within broader debates on OS, this exploratory research contributes to a deeper understanding of how disciplinary and institutional contexts shape adoption, while also identifying priority areas for future investment and research.

## Conclusion

This study provides institution-specific evidence on the adoption of OS practices within a Portuguese nursing school. The survey-based approach revealed that while open access publishing is relatively well accepted, other practices, such as preregistration of studies, open-source code, and systematic data sharing, remain unfamiliar and underutilized. Participants expressed alignment with the principles of OS, yet persistent gaps were observed in participants’ awareness and knowledge of institutional and international policies.

These findings carry significant implications for HEI. They highlight the necessity of designing training initiatives that extend beyond open access to encompass the full spectrum of OS practices, strengthen institutional support mechanisms, and establish incentive systems that reward openness. Furthermore, results indicate that nursing and health sciences require tailored strategies that reconcile the principles of openness with ethical and confidentiality obligations. Effectively addressing these challenges can foster a culture of trust, collaboration, and transparency within the academic community.
