# Correction: Open Science practices in a Portuguese nursing higher education institution: An exploratory, descriptive study

**DOI:** 10.1371/journal.pone.0342028

**Published:** 2026-01-30

**Authors:** Ana Sabrina Sousa, Mafalda Lopes, Diana Rodrigues, Palmira Oliveira, Regina Pires, Sara Pinto, Pedro Melo, João Frias Rosa, Amparo Alves, Rosa Silva

The ORCID iDs are missing for the third, fifth, sixth, eighth, ninth and tenth authors. Please see the authors’ respective ORCID iDs here:

- Author Diana Rodrigues’s ORCID iD is: 0000-0002-7602-1313

(https://orcid.org/0000-0002-7602-1313).

- Author Regina Pires’s ORCID iD is: 0000-0003-1610-7091

(https://orcid.org/0000-0003-1610-7091).

- Author Sara Pinto’s ORCID iD is 0000-0001-9327-5270

(https://orcid.org/0000-0001-9327-5270).

- Author João Frias Rosa’s ORCID iD is 0000-0001-6328-9807

(https://orcid.org/0000-0001-6328-9807).

- Author Amparo Alves’s ORCID iD is 0009-0009-9178-8713

(https://orcid.org/0009-0009-9178-8713).

- Author Rosa Silva’s ORCID iD is 0000-0002-3947-7098

(https://orcid.org/0000-0002-3947-7098).
